# Loss of IgA and IgM Compromises Broad Neutralization of Structurally Divergent SARS-CoV-2 Variants

**DOI:** 10.3390/antib14030059

**Published:** 2025-07-12

**Authors:** Yalcin Pisil, Tomoyuki Miura, Kiyoki Ito, Yoshihiro Watanabe

**Affiliations:** 1 Laboratory of Primate Model, Research Center for Infectious Diseases, Institute for Life and Medical Science, Kyoto University, Kyoto 615-8530, Japan; 2 Graduate School of Medical Sciences, Kanazawa University, Kanazawa 920-0934, Japan; kiyokiyo1980fukui@yahoo.co.jp (K.I.); yoshi.watanabe@staff.kanazawa-u.ac.jp (Y.W.)

**Keywords:** SARS-CoV-2, mAb, neutralization, IgA, IgG, IgM, lambda, omicron, vaccine response, luciferase

## Abstract

**Objectives:** The durability and breadth of neutralizing antibodies following SARS-CoV-2 mRNA vaccination remain incompletely understood. This study aimed to investigate how longitudinal changes in antibody isotype composition impact neutralization against structurally diverse SARS-CoV-2 variants. **Methods:** After screening a broader cohort of mRNA-vaccinated sera, time-matched samples collected one month (1 mpv) and three months post-vaccination (3 mpv) were selected for detailed analysis. Neutralization assays against live virus variants, enzyme-linked immunosorbent assays (ELISA), and immunogold electron microscopy were performed to assess antibody titers, isotype levels, and virion morphology. **Results:** Neutralization titers declined markedly at 3 mpv, particularly against immune-evasive variants. Notably, the Lambda variant showed disproportionately high sensitivity to early-phase sera despite its divergence from the vaccine strain. Antibody isotyping showed that IgA and IgM decreased over time, while IgG levels were relatively more sustained. Electron microscopy revealed broader virion size heterogeneity in Lambda (50–200 nm) compared to Wuhan (80–120 nm), potentially enhancing multivalent antibody engagement. Consistently, ELISA under reduced spike density conditions showed that IgA and IgM retained stronger binding than IgG. **Conclusions:** These findings indicate that the decline of IgA and IgM compromises neutralization breadth, especially against structurally divergent variants such as Lambda. Sustaining dynamic multivalent isotype responses that adapt to diverse spike morphologies may be critical for broad cross-variant immunity.

## 1. Introduction

The initial antibody response to SARS-CoV-2 is typically immunoglobulin M (IgM), detectable within the first week of symptoms. IgM levels surge to their highest point approximately 2 weeks after the onset of disease symptoms, gradually declining after the 1st month and disappearing in 2–3 months. On the other hand, immunoglobulin G (IgG) levels reach their peak one month after the appearance of symptoms and persist for up to three months [1,2]. Immunoglobulin A (IgA) production follows IgM, becoming detectable around day 11 and peaking 3–4 weeks post-infection [3,4,5]. IgA response is stronger and more persistent than the IgM response [5].

Similarly, to infection, vaccines result in the early production of serum IgA, IgM, and IgG antibodies and also induce long-term memory B and T cell responses [6,7]. Currently, IgG mAbs dominate the therapeutic antibody field due to their prolonged plasma half-life and their effective activation of complement, along with the recruitment of Natural Killer cells for antibody-dependent cellular cytotoxicity (ADCC). IgA responses are more effective when active or passive mucosal immunity is needed, compared to IgG. Given that SARS-CoV-2 primarily infects through the respiratory mucosa, enhancing mucosal immunity through IgA may offer an additional layer of defense not achievable by IgG-centric approaches. Moreover, IgA mAbs excel in recruiting neutrophils to kill tumor cells through antibody-mediated mechanisms, offering a potential solution to combat apoptosis resistance in these cells. The common misinterpretation of IgA as a non-inflammatory antibody needs correction, as not many researchers have recognized its potent inflammatory role in specific circumstances [8,9].

Unlike IgG, which is bivalent, IgM forms pentamers and IgA can form dimers with secretory components, which are features that provide increased avidity and enable multivalent antigen engagement [10,11,12,13]. This structural advantage likely contributes to their superior neutralization efficiency. Importantly, even when antibodies are engineered to share the same antigen-binding region (Fab domain), IgA and IgM have been shown to exhibit dramatically enhanced in vitro neutralization potency compared to IgG. In our previous work, both isotypes demonstrated up to 125- and 225-fold greater neutralization, respectively, than their IgG counterparts when tested against SARS-CoV-2 pseudovirus [14,15]. These findings are consistent with Ku et al. 2021, who demonstrated that converting existing anti-SARS-CoV-2 IgG clones into IgM significantly restored neutralization activity against SARS-CoV-2 variants [16]. For instance, memory B cell-derived antibodies, re-expressed in IgM format with unchanged variable regions, exhibited ~15-fold stronger neutralizing activity than their IgG versions against multiple SARS-CoV-2 variants [17]. In another study, Markotte et al. (2024) demonstrated that dimeric IgA and secretory dimeric IgA bearing the same Fab domain exhibited substantially greater neutralization potency against Omicron variants than either monomeric IgA or IgG [18]. Similarly, secretory dimeric IgA engineered with the same VH/VL domains as potent IgG clones significantly outperformed IgG in neutralizing Omicron BA.1 and BA.2 [19].

Given the dynamic nature of isotype responses following infection or vaccination, and the distinct effector functions mediated by IgG, IgA, and IgM, it is critical to understand how each contributes to functional immunity in vivo. To investigate this, we analyzed human sera with high neutralizing capacity against SARS-CoV-2 to determine whether the observed efficacy was primarily due to IgG or due to elevated levels of IgA or IgM. We performed isotype-specific enzyme-linked immunosorbent assay (ELISA) targeting the SARS-CoV-2 Wuhan strain spike protein and complemented this analysis with negative-stain electron microscopy (NS-EM) to visualize antibody binding at the ultrastructural level. This combined approach allowed us to dissect the potential contributions of antibody isotype composition to viral neutralization in a physiologically relevant context.

Despite the wealth of literature on SARS-CoV-2 antibodies and vaccines, few studies have systematically dissected how the waning of IgA and IgM—despite their demonstrated in vitro potency—affects long-term cross-variant neutralization breadth in human sera after mRNA vaccination. Given the continual emergence of immune-evasive variants and the limitations of IgG-centric strategies, understanding the dynamics and decay of multivalent isotype responses remains highly relevant. Our study directly addresses this gap by combining serological profiling, variant-specific neutralization assays, and ultrastructural imaging, offering new insights into the functional implications of isotype decline beyond what has been shown in prior work.

## 2. Materials and Methods

### 2.1. Viruses

All experiments involving live SARS-CoV-2 variants were conducted in biosafety level 3 (BSL-3) facilities at the Experimental Research Center for Infectious Diseases, Institute for Life and Medical Sciences, Kyoto University, Japan. SARS-CoV-2 variants were kindly provided by Dr. Michio Suzuki from the Department of Veterinary Science, National Institute of Infectious Diseases, Ministry of Health, Labour and Welfare, Toyama Building, Japan (Table 1). Vero E6 cells were cultured as previously described [20,21,22]. The virus was passaged in Vero E6 cells to generate a working stock (BSL-3 stock), which was used in all experiments. Fully confluent Vero E6 monolayers were inoculated with virus and maintained in Dulbecco’s Modified Eagle Medium (DMEM) supplemented with 2% fetal calf serum (FCS), 2 mM L-glutamine, and G418. TCID_50_ values were calculated based on cytopathic effect (CPE) observations performed as previously described [23], using Monte Carlo simulations based on the Spearman–Kärber method, as previously defined [22] (Table 1). Serial dilutions were performed starting at 1:10 with a 10-fold dilution factor, applying 200 µL per well and using six replicate wells per dilution.

### 2.2. Cell Culture

HEK293T cells (American Type Culture Collection (ATCC) CRL 3216) and CRFK cells (ATCC CCL-94) were cultured in Dulbecco’s Modified Eagle Medium (DMEM) (Fujifilm Wako Pure Chemical Corporation, Osaka, Japan), supplemented with 10% (*v*/*v*) heat-inactivated fetal bovine serum, 2 mM sodium pyruvate (MP Biomedicals Inc., Santa Ana, CA, USA) and 4 mM L-glutamine (Fujifilm Wako Pure Chemical Corporation). African green monkey kidney clone of Vero-E6 (VeroE6) cells (ATCC CRL 1586) were maintained in Dulbecco’s Modified Eagle Medium (DMEM) supplemented with 5% fetal bovine serum (FBS), 2% L-glutamine, and 1 mg/mL G418. The complete medium was prepared as follows: DMEM with 5% FBS (2.5 mL), 2% L-glutamine (1 mL), G418 (1 mg/mL) (500 µL from 1 mg/mL stock solution). Cells were harvested and passaged using trypsin/ethylenediaminetetraacetic acid solution (Nacalai Tesque, Kyoto, Japan). All cell lines were maintained at 37 °C under 5% CO_2_ in a humidified environment. CPE was observed as previously described [24]. SARS-CoV-2-vaccinated human sera were initially diluted 1:30 and then subjected to 2-fold serial dilutions across 9 wells. The 10th well served as a control. SARS-CoV-2 variants were added to each dilution tube at a calculated TCID_50_ dose. Each 50 µL of virus was mixed with 100 µL of diluted serum and incubated at 37 °C for 1 h. After incubation, the mixture was transferred onto fully confluent Vero E6 monolayers in 96-well plates. The plates were incubated at 37 °C with 5% CO_2_ for 3 days, after which CPE was assessed by light microscopy.

### 2.3. Enzyme-Linked Immunosorbent Assay (ELISA) of mAbs

Elisa experiments were designed as described previously [25]. Commercial COVID-19 Human IgM/IgG ELISA kits (Full Spike (Wuhan) Protein) (ERCOEL961) and COVID-19 Human IgA ELISA kit (Full Spike (Wuhan) Protein) (RCAEL961-SF) were purchased from Cellspect. SARS-CoV-2 Spike (Wuhan) Protein Serological IgA ELISA Kit #58873C, SARS-CoV-2 Spike (Wuhan) Protein Serological IgM ELISA Kit #37322C, and SARS-CoV-2 Spike (Wuhan) Protein Serological IgG ELISA Kit #20154C were purchased from Cellspect Signaling, distributed by Osaka Yaken Co., Ltd. (Osaka, Japan). ELISA assays were conducted according to each manufacturer’s recommendations. ELISA plates coated with full spike were first washed twice with PBS containing 0.05% Tween 20 (PBS-T) and subsequently blocked with PBS-T supplemented with 5% skim milk for 1 h at room temperature.

Subsequently, 100 µL samples of sera were serially diluted in diluent buffer containing 2% non-fat milk. The diluted sera were then incubated overnight at 4 °C. The plates were subsequently washed five times with PBS-T, and 100 µL of horseradish peroxidase (HRP)-conjugated secondary antibodies were added: Anti-Human IgG and IgM (Cellspect), diluted by 1:30,000 from stock Anti-Human IgA (Cellspect), diluted by 1:20,000. Plates were incubated at room temperature for 2 h. Following incubation, the reaction solution was discarded, and the plates were washed five times with PBS-T. To initiate the colorimetric reaction, 100 µL of TMB solution—pre-warmed to room temperature—was added to each well. The plates were then gently shaken for 6 min at room temperature. The reaction was stopped by the addition of stop solution. Optical density at 450 nm (OD_450_) was measured using a TriStar LB 941 microplate reader (Berthold Technologies, Bad Wildbad, Germany) with MikroWin 2000 version 4.41 English UI software.

### 2.4. Generation of Pseudo-Typed Lentivirus with SARS-CoV-2 S Particles

Cloning of all DNA plasmids was performed by transformation into Escherichia coli Stbl3 cells, as previously described [26]. Lentiviral-based pseudo-typed viruses were generated as described in prior studies [27]. Pseudo-typed lentiviruses coated with the SARS-CoV-2 S protein were produced using the 2019-nCoV_pcDNA3.1(+) P2A-eGFP spike vector (1 µg/mL) by co-transfecting 5 × 10^5^ HEK293T cells/mL with the ‘HIV-1 NL4-3 ΔEnvΔVpr Luciferase Reporter Vector (1 µg/mL)’ [28]. Additionally, 200 µL of CTS Opti-MEM (Product number: A4124801) and 7 µL of X-Treme HP transfection reagent were added to facilitate transfection. Transfected HEK293T cells were incubated at 37 °C under 5% CO_2_ for 48 h, after which the supernatant containing pseudo-typed lentivirus particles coated with SARS-CoV-2 S protein was harvested and filtered through a 0.45 µm filter (Millipore, Burlington, MA, USA). Aliquots of 400 µL were stored in 1.5 mL tubes at −80 °C for further experiments. The HIV-1 NL4-3 ΔEnvΔVpr Luciferase Reporter Vector (pNL4-3.Luc.R-E-) (catalog number 3418) was provided by Dr. Nathaniel Landau (NIH AIDS Reagent Program, Division of AIDS). The 2019-nCoV_pcDNA3.1(+) P2A-eGFP spike vector (catalog number MC_0101087) was obtained from Nacalai Tesque via Molecular Cloud. Expression of eGFP in transfected 293T cells was verified using an EVOS FL AMF 4300 microscope (Thermo Fisher Scientific) with excitation/emission settings as follows: Ex: 470/22 nm, Em: 525/20 nm.

### 2.5. Generation of ACE2 and TMPRSS2-Transfected CRFK Cells

CRFK cells were seeded at a density of 5 × 10^5^ cells/mL in six-well plates using DMEM supplemented with 10% fetal bovine serum (FBS), 2% L-glutamine, and 1% pyruvate. The cells were incubated overnight until they reached 80–90% confluency. Transfection was performed using 1 µg/mL pcDNA3.1+/C-(K) DYK-ACE2 plasmid**,** 1 µg/mL TMPRSS2 expression plasmid**.** The plasmids were mixed with 200 µL CTS Opti-MEM (Product number: A4124801) and 7 µL X-TremeGENE HP transfection reagent, following the manufacturer’s instructions. The transfected cells were incubated at 37 °C with 5% CO_2_ for 24 h. The pcDNA3.1+/C-(K) DYK-ACE2 plasmid (catalog number MC_0101086) was obtained from Molecular Cloud via Nacalai Tesque. The TMPRSS2 expression plasmid (catalog number #53887) was purchased from Addgene via Osaka Yaken Co.

### 2.6. Neutralization Assays

Neutralization assays were performed using adjusted viral doses to ensure equivalent infectivity levels across all cell lines. The assays were conducted in a 96-well format, following previously established protocols. The ID_50_ value was defined as the sample dilution or antibody concentration at which relative luminescence unit (RLU) readings were reduced by 50% compared to virus control wells (cells plus virus without test sample), after subtracting background RLU values from cell control wells (cells only, no virus or test sample) [29]. Human sera were kindly obtained from Kanazawa University Hospital (Japan) via Dr. Yoshihiro Watanabe and Dr. Kiyoaki Ito, with informed consent and institutional ethical approval as described in the Ethics Statement section. SARS-CoV-2 plasma serum of COVID-19 patients with varying IgM and IgG antibody levels were purchased from RayBiotech (Peachtree Corners, GA, USA). The human serum sample was subjected to serial two-fold dilutions. Then, 50 µL of pseudo-typed virus at a concentration of 6000 RLU mL^−1^ was added to wells containing the diluted plasma. Control wells contained no plasma, only cells and virus. The plasma–virus mixtures were incubated at 37 °C under 5% CO_2_ for 1 h. After incubation, ACE2-TMPRSS2-expressing CRFK cells seeded in 96-well plates. The plates were incubated at 37 °C under 5% CO_2_ for 2 days, and then the luciferase activity of the samples was measured. The experimental procedure for testing neutralizing mAbs was identical to the method used for assessing plasma neutralization ability. All mAbs were purchased from Elabscience (Houston, TX, USA)**.** The luciferase-based neutralization assay was performed as previously described in 30, 31, 32, 59. Briefly, 50 µL of cell lysate solution (Toyo B-Net, Tokyo, Japan) was added to each well, followed by agitation for 15 min. A 30 µL aliquot of lysate was transferred to a Nunc F96 MicroWell white plate (Thermo Fisher Scientific, Waltham, MA, USA). An amount of 30 µL of luminescent substrate was added to each well. Luciferase activity was measured using a TriStar LB 941 microplate reader (Berthold Technologies, Bad Wildbad, Germany) with MikroWin 2000 version 4.41 English UI software.

### 2.7. Immuno-NS-EM

The NS-EM protocol was adapted from an optimized negative-stain method [30] and performed to visualize the binding of human antibodies to authentic SARS-CoV-2 particles. Viral preparations were incubated with human sera (1 mpv or 3 mpv) or anti-SARS-CoV-2 mAbs for 1 h at 37 °C, followed by labeling with gold-conjugated Anti-Human IgG, IgA, or IgM secondary antibodies.

Three anti-SARS-CoV-2 mAbs were used, including Human IgG (Catalogue number E AB V1021), Human IgM (Catalogue number E AB V1026), Human IgA (Catalogue number E AB V1027). All mAbs belonged to the 8A5 clone Fab, which was elicited using recombinant 2019 nCoV S trimer Protein (His Tag) as the immunogen. All mAbs were purchased from Elabscience (Houston, TX, USA).

Three 4 nm Colloidal Gold AffiniPure Goat Anti-Human Ab were used:

4 nm Colloidal Gold-AffiniPure Goat Anti-Human IgM, Fc5 Fragment Specific (LM Grade) (Wakenyaku-Jackson ImmunoResearch Laboratories, Product Code: 109-185-043).

4 nm Colloidal Gold-AffiniPure Goat Anti-Human IgG, Fc gamma Fragment Specific (min X Bov, Ms, Rb Sr Prot) (Wakenyaku-Jackson ImmunoResearch Laboratories, Product Code: 109-185-170).

4 nm Colloidal Gold-AffiniPure Goat Anti-Human Serum IgA, α Chain Specific (LM Grade) (Nacalai-Jackson ImmunoResearch Laboratories, Inc., Product Code: 109-185-011).

A 2.5% glutaraldehyde solution and 2% (*w*/*w*) phosphotungstic acid (PTA) solution were prepared following the Electron Microscope Unit, University of Hong Kong Sample Preparation Techniques for electron microscopy protocol. Viruses were inactivated in a 1:1 mixture of 2.5% glutaraldehyde and 0.1 M sodium cacodylate-HCl buffer (pH 7.4) at room temperature for 1 h in BSL3 lab. A 3 µL aliquot of inactivated samples was applied onto glow-discharged grids coated with a continuous carbon layer and incubated for 1 min. Excess liquid was blotted using filter paper to remove residual sample. The grids were then immediately stained with 2% (*w*/*w*) PTA, and redundant stain was removed using filter paper. Air-dried grids were imaged at 120 keV using a JEOL 1200EX transmission electron microscope at 120K magnification.

### 2.8. ELISA for Binding Dynamics of mAbs at Varying Spike Protein Concentrations

Recombinant trimeric SARS-CoV-2 spike protein (His-tagged; CST #16-1208) was obtained via Wakenyaku, Japan. ELISA plates (Thermo Fisher Maxisorp, Cat# 439454) were coated with serially diluted spike protein in PBS at concentrations of 1000, 300, 100, and 30 ng/mL, and incubated at 4 °C for 48 h. After washing and blocking, serially diluted human monoclonal IgG, IgA, and IgM antibodies were applied and incubated overnight at 4 °C. Plates were then treated with HRP-conjugated secondary antibodies specific to each isotype, followed by TMB substrate development. Absorbance was measured at 450 nm using a plate reader.

## 3. Results

### 3.1. Assessment of SARS-CoV-2 Variant Neutralization by Human Sera 3–6 Months After mRNA Vaccination

In our previous study, we established a neutralization assay using lentiviral-based pseudo-typed virus with SARS-CoV-2 spike protein (LpVspike) in ACE2-expressing CRFK cells to assess the neutralizing efficacy of monoclonal antibodies and commercial sera [14]. In that assay, commercial IgM-containing sera exhibited significantly higher neutralization activity against LpVspike compared to IgG-containing sera. Moreover, monoclonal antibodies of the IgA and IgM isotypes, sharing the same Fab domain, demonstrated greater neutralizing potency than their IgG counterparts [14].

**Figure 1:**
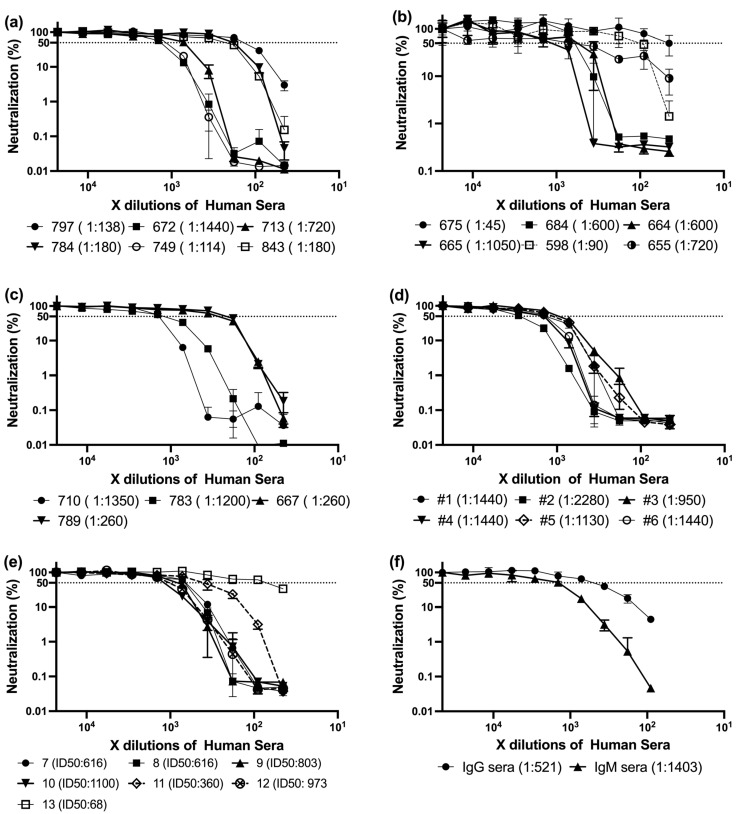
Neutralization activity of mRNA-vaccinated sera against LpVspike. Each panel shows neutralization curves for a group of 5–7 sera with varying neutralization potency. Sera were serially diluted and tested in our neutralization assay system. ID50 values were calculated using non-linear regression. These representative data illustrate the variability in neutralizing antibody responses across different individuals. Experiments were performed in triplicate, and error bars represent the standard deviation; median values are indicated. (**a**) ID50 neutralization titers of vaccinated human sera (Samples no. 797,672, 713, 784, 749, 843. (**b**) ID50 neutralization titers of vaccinated human sera (Samples no. 675, 684, 664, 665, 598, 655. (**c**) ID50 neutralization titers of vaccinated human sera (Samples no.710, 783, 66, 789). (**d**) ID50 neutralization titers of vaccinated human sera (Samples no.#1, #2, #3, #4, #5, #6). (**e**) ID50 neutralization titers of vaccinated human sera (Samples no. 7, 8, 9, 10, 11, 12). (**f**) ID50 neutralization titers of convalescent human sera (IgG dominant, IgM dominant samples).

Building upon these findings, we analyzed a panel of sera collected from individuals who had received three doses of mRNA-based SARS-CoV-2 vaccines. All samples were obtained approximately three to six months after the third dose, corresponding to a period of waning immunity. Utsunomiya-Higashi Hospital kindly provided these sera, which were obtained from healthcare professionals and hospital staff, including nurses, medical staff, and administrative personnel. According to the sample providers, these individuals had definitively received at least three vaccine doses, and many had a relatively high probability of prior SARS-CoV-2 infection, which may have further influenced their immune profiles (Figure 1a–e).

**Figure 2:**
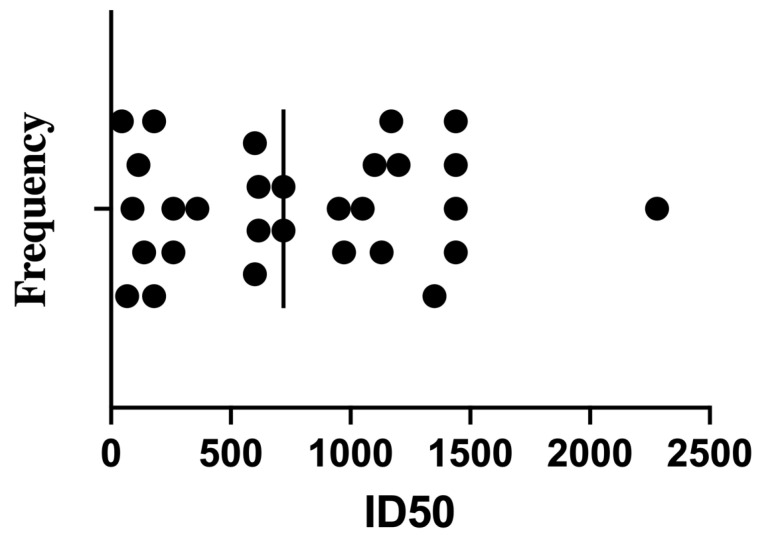
Frequency distribution of LpVspike neutralization titers (ID50) in mRNA-vaccinated human sera. Each bar represents the number of samples falling within a given ID50 range. Data were derived from the ID50 values presented in Table 2 and Figure 1, which were calculated based on LpVspike neutralization assays. This figure highlights the heterogeneity of neutralizing responses in vaccinated individuals.

Despite the shared vaccination history, the neutralization assay revealed substantial inter-individual variation in ID_50_ titers (the serum dilution required to inhibit 50% of viral infectivity). Subsequently, the ID50 was calculated as described earlier [31]. Titers ranged broadly from low to very high values (Table 2, Figure 2). The frequency distribution showed most samples fell between ID_50_ values of 200 and 1200, with a median around 720. Notably, only a few sera exceeded ID_50_ > 1500—for example, sample #2 reached 2280—potentially reflecting robust memory responses in individuals with hybrid immunity (vaccination + prior infection).

These high-titer samples may serve as valuable candidates for further investigation into antibody isotype profiles and epitope targeting. Conversely, the fact that many participants exhibited neutralizing activity below the cohort median, despite having received booster vaccination 3–6 months prior, underscores the heterogeneity of post-vaccination immune durability.

Collectively, these results suggest that even after a third vaccine dose, a substantial proportion of individuals—especially those without confirmed prior infection—may display relatively modest neutralization capacity, reinforcing the need to assess not only vaccine schedule, but also infection history and isotype-driven quality of the antibody response.

These findings formed the basis for selecting representative sera for further characterization of antibody isotype composition and neutralization profiles.

### 3.2. Serological Profiling of SARS-CoV-2 Spike-Specific Antibody Isotypes (IgG, IgA, IgM) Following mRNA Vaccination

To evaluate the humoral immune profiles elicited by mRNA vaccination, we performed serological analyses of SARS-CoV-2-specific antibody isotypes (IgG, IgA, and IgM) using ELISA. Serum samples were primarily selected based on high ID_50_ neutralization titers from the pseudovirus assay and were supplemented with medium- and low-ID_50_ samples to allow comparative analysis across a broad activity range (Table 2).

**Figure 3:**
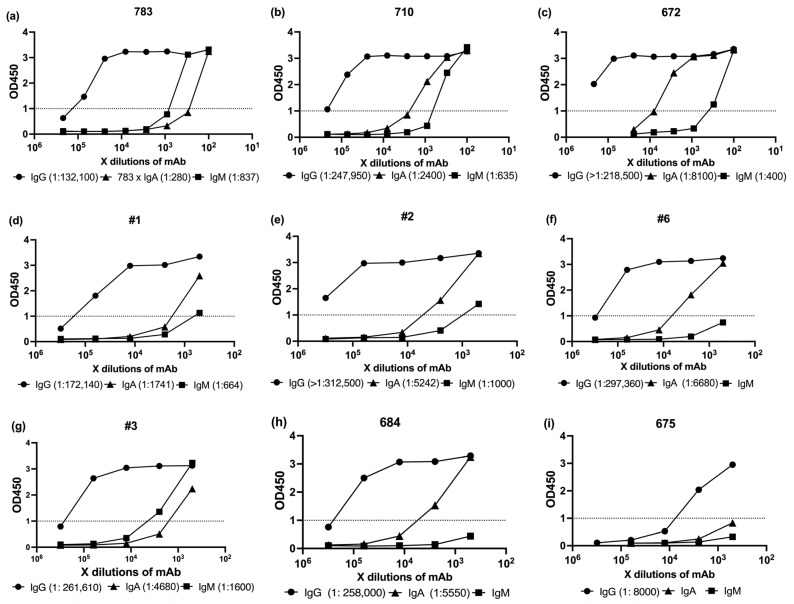
Isotype-specific ELISA binding curves for anti-SARS-CoV-2 spike protein IgG, IgA, and IgM antibodies present in vaccinated human sera. Each curve represents the binding signal (OD_450_) obtained using HRP-conjugated Anti-Human IgG, IgA, or IgM secondary antibodies. The antibody dilution required to reach OD_450_ = 1.0 is indicated in parentheses, serving as a relative indicator of binding strength. To compare the relative abundance and binding strength of antibody isotypes, we selected OD_450_ = 1.0 as a standardized reference point. Antibodies reaching this signal at lower serum dilutions are interpreted to have stronger or more abundant binding to the spike protein under these assay conditions. (**a**–**f**) Serological Profiling of Higher ID50 sera, (**g**–**i**) Serological Profiling of Intermediate and lower ID50 sera.

For this purpose, we employed commercially available ELISA kits targeting the full-length SARS-CoV-2 spike protein. IgG, IgA, and IgM kit were purchased from Cellspect or Cell Signaling Technology. Detailed procedures and kit specifications are provided in Section 2. All assays were conducted strictly in accordance with the manufacturers’ protocols (Figure 3).

**Figure 4:**
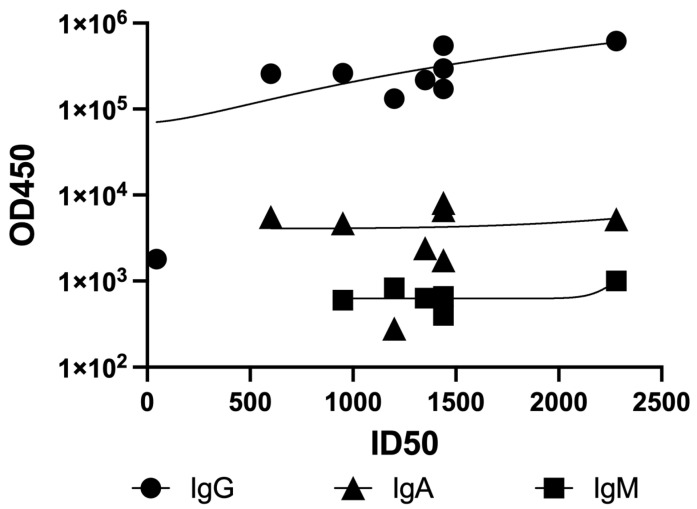
Correlation Between neutralization titers (ID50) and SARS-CoV-2 spike-specific antibody levels in vaccinated sera. Correlation analysis between neutralization titers (ID50) and antibody levels measured by ELISA (OD_450_) against SARS-CoV-2 spike protein (Wuhan strain). *Y*-axis is plotted on a logarithmic scale to better visualize changes across different antibody isotypes. Pearson correlation coefficients (r) and corresponding p-values are shown separately for each antibody isotype: IgG (r = 0.017, *p* > 0.05, not significant), IgA (r = 0.085, *p* > 0.05, not significant), IgM (r = 0.096, *p* > 0.05, not significant).

To compare antibody levels with neutralization potency, we first measured spike-specific IgG, IgA, and IgM by ELISA (Figure 3). We then compiled the ID50 titers (Table 2) and ELISA OD1 values into Table 3, which served as the basis for the correlation analysis shown in Figure 4.

In vaccinated sera, no statistically significant correlation was observed between neutralization titers (ID50) and antibody levels measured by ELISA against the SARS-CoV-2 Wuhan strain spike protein (Figure 4). Pearson correlation coefficients were notably weak: r = 0.017 for IgG, r = 0.085 for IgA, and r = 0.096 for IgM.

This lack of correlation led us to consider two possible explanations. First, Fc-mediated effector functions such as ADCC and ADCP may have contributed to viral clearance in vivo, although these mechanisms were not directly assessed in our study and thus remain speculative [32,33]. Second, differences in the timing of serum collection and the associated shifts in isotype composition could have influenced neutralization potency independently of total antibody levels. To explore this possibility, we collected sera at defined intervals after vaccination to enable a more controlled evaluation of antibody kinetics. IgG levels typically peak between two weeks and one month after infection or vaccination and remain detectable for up to 90 days, whereas IgM levels decline significantly after the first month [1]. By analyzing samples collected at different timepoints, we aimed to investigate how temporal changes in isotype composition may affect neutralization capacity.

### 3.3. Early Post-Vaccination Sera Exhibit Superior Neutralization of SARS-CoV-2 Pseudovirus

Before presenting the results of these temporally stratified vaccination sera, we first re-examined prior data comparing IgM-rich and IgG-rich convalescent samples to provide a contextual baseline. We previously determined the ID50 values of two types of COVID-19 convalescent sera with differing IgM and IgG profiles against LpVspike [14]. These findings suggested that IgM-enriched sera may be more potent in neutralizing the LpVspike than their IgG-dominant counterparts. Building on this observation, our current analysis revealed a similar trend: the ID50 ratio between representative IgM-rich and IgG-rich convalescent sera was approximately 2.7-fold (Figure 1f), reinforcing the hypothesis that multivalent IgM plays a critical role in serum neutralization. This concordance between independent datasets strengthens the mechanistic model in which isotype composition—not merely total antibody levels—significantly influences neutralization efficacy, particularly during the early immune response.

To further investigate the dynamics of isotype-driven neutralization, we next examined serum samples collected from individuals one month (1 mpv) and three months (3 mpv) after receiving a SARS-CoV-2 spike-based mRNA vaccine. This approach allowed us to evaluate how the balance of IgG, IgA, and IgM antibodies evolves over time and contributes to functional immunity following vaccination.

**Figure 5:**
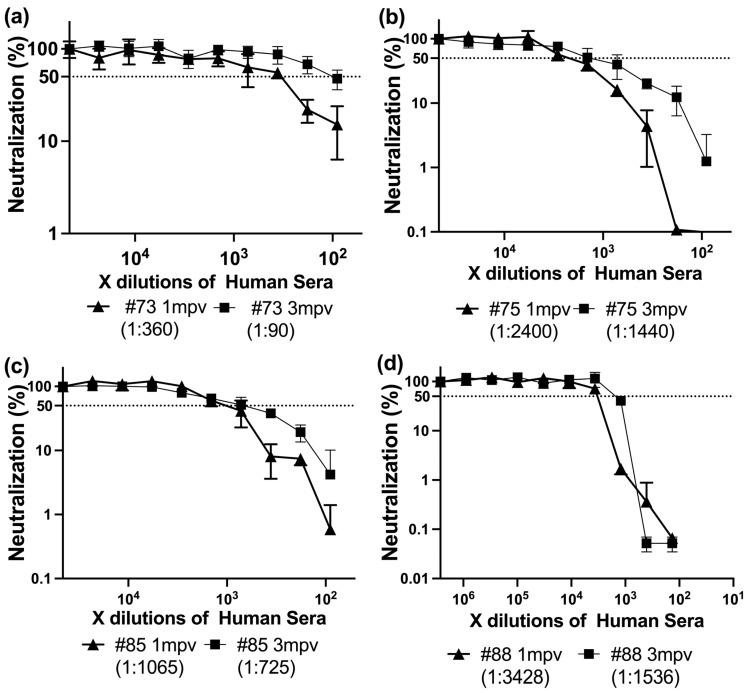
Neutralizing activity of 1mpv and 3mpv mRNA-vaccinated sera against LpVspike. Serial dilutions of human sera collected 1 mpv and 3 mpv after at least two doses of vaccination for #73, #75, #83 and least three doses for #88 were tested for their ability to neutralize LpVspike. The virus-sera mixtures were incubated with ACE2/TMPRSS2-transfected CRFK cells for 48 h, and luciferase activity was measured as an indicator of infection. Neutralization curves were generated from serial 2-fold dilutions (starting at 1:90), and ID50 values were calculated. (**a**) ID50 neutralization titer of vaccinated human sera (#73) collected 1mpv and 3 mpv. (**b**) ID50 neutralization titer of vaccinated human sera (#75) collected 1mpv and 3 mpv. (**c**) ID50 neutralization titer of vaccinated human sera (#83) collected 1mpv and 3 mpv. (**d**) ID50 neutralization titer of vaccinated human sera (#88) collected 1mpv and 3 mpv.

To evaluate the effect of time post-vaccination on neutralizing antibody responses, we analyzed sera from four individuals collected at 1 mpv and 3 mpv following SARS-CoV-2 spike vaccination (either Moderna or BNT162b2). All 1 mpv sera exhibited significantly higher neutralizing titers (ID50 range: 360–3428) compared to their 3 mpv counterparts (ID50 range: 90–1536) (Figure 5).

In randomly collected vaccinated sera, correlation analysis revealed no significant association between IgG, IgA, or IgM levels (OD_450_) and neutralization titers (ID50), likely reflecting inter-individual variability in timing of infection and immune maturation. In contrast, in time-matched sera collected 1 mpv and 3 mpv, declining IgA and IgM levels were clearly associated with decreased neutralizing potency. This temporal alignment supports the interpretation that it is changes in isotype composition, rather than absolute antibody levels, that critically influence neutralizing efficacy.

### 3.4. Temporal Changes in Antibody Isotypes Reveal IgA and IgM Contribution to Neutralization Potency After mRNA Vaccination

To investigate the reason behind the stronger neutralizing capacity of 1 mpv sera compared to 3 mpv sera, we analyzed antibody isotypes and their relative levels using ELISA. Elisa assays are described in Section 2.

**Figure 6:**
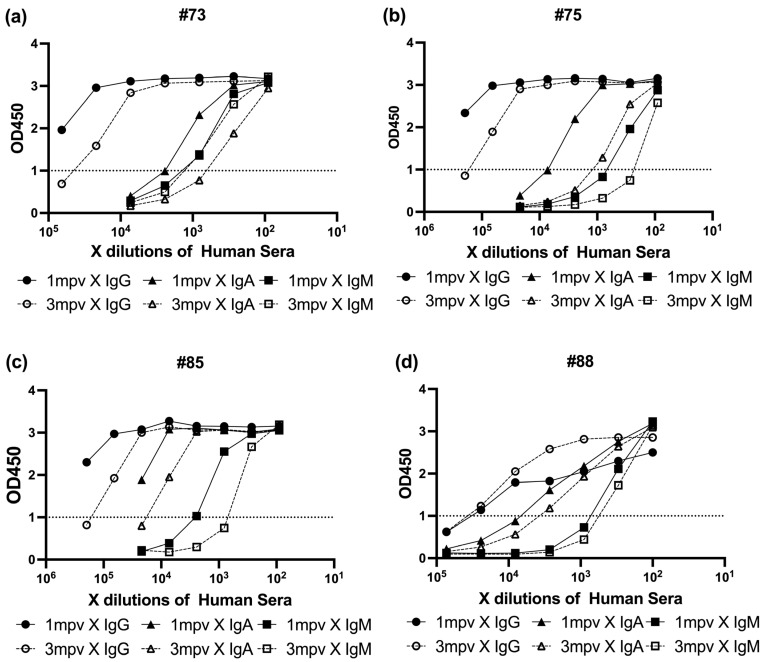
Comparison of SARS-CoV-2 spike-specific antibody levels in vaccinated human sera by ELISA. Serial dilutions of sera from vaccinated individuals collected at 1 mpv and 3 mpv were tested by ELISA against SARS-CoV-2 full spike protein. Anti-Human IgG, IgA, and IgM antibodies conjugated with horseradish peroxidase (HRP) were used for detection. Optical density at 450 nm (OD_450_) was measured to assess the binding of spike-specific antibodies in each sample. (**a**) Isotype determination in serum samples from donor #73 collected at 1 mpv and 3 mpv. (**b**) Isotype determination in serum samples from donor #75 collected at 1 mpv and 3 mpv. (**c**) Isotype determination in serum samples from donor #85 collected at 1 mpv and 3 mpv. (**d**) Isotype determination in serum samples from donor #88 collected at 1 mpv and 3 mpv.

To determine the relationship between neutralizing activity and spike-specific antibody isotype levels, we performed ELISA on sera collected 1 mpv and 3 mpv (Figure 6). Spike protein-coated ELISA plates were incubated with serially diluted sera, followed by detection with HRP-conjugated Anti-Human IgG, IgA, or IgM antibodies. Absorbance at 450 nm (OD450) was recorded to assess the binding levels of each isotype. In each plot, black symbols represent 1 mpv samples and white symbols represent 3 mpv (●/○: IgG, ▲/△: IgA, ■/□: IgM) (Figure 6).

All four individuals exhibited a common isotype hierarchy, with IgG responses dominating, followed by IgA, and IgM showing the lowest binding signal. However, the patterns of decay varied across individuals and timepoints.

#73 showed a largest decline in neutralizing titer (4-fold) and ELISA absorbance values, with IgG and IgA each decreasing by approximately half a log, while IgM levels remained relatively stable (Figure 6a).

In #75, which experienced only a 1.7-fold drop in neutralization potency, IgG and IgA levels each declined by roughly one log, while IgM showed a smaller decrease of about half a log (Figure 6b).

For #85, the overall reduction in neutralizing activity was minimal (1.5-fold), and all three isotypes—IgG, IgA, and IgM—showed modest declines of about half a log each (Figure 6c).

Interestingly, #88 exhibited the second largest drop in neutralization titer (2.2-fold), yet IgG binding remained stable over time, with only slight decreases observed in IgA and IgM signals (Figure 6d).

These results collectively suggest that while IgG is the predominant isotype contributing to spike binding, subtle shifts in IgA and IgM levels—particularly in individuals with previously strong neutralizing activity—can still contribute meaningfully to changes in overall neutralization potency. This underscores the importance of considering isotype composition, not just total antibody levels, when evaluating vaccine-induced humoral immunity over time.

### 3.5. Early Broad Neutralization of SARS-CoV-2 Variants Highlights Exceptional Sensitivity of Lambda and Justifies Structural Comparison with Wuhan

The timepoints 1 mpv and 3 mpv of #75, #85 and #88 sera had highest neutralizing activity against LpVspike pseudovirus (Figure 5). To evaluate the neutralizing capacity of vaccine-induced sera wanes not only in potency but also in breadth over time, we performed live-virus neutralization assays using serum from these vaccinated individuals (#75, #85 and #88) against a panel of authentic SARS-CoV-2 variants.

The neutralization activity of #75, #85 and #88 sera collected at 1 mpv and 3 mpv were tested against 16 SARS-CoV-2 strains, including the original Wuhan strain and variants from Alpha, Beta, Gamma, Epsilon, Lambda, Theta, Mu, and multiple Omicron sublineages (BA.1.18, BA.1.1, BA.4, BA.5, XE, XBB.1, and BA.5.11111). Neutralization titers (ID50) were determined for each timepoint and strain (Table 4). Overall, sera collected at 1 mpv exhibited significantly higher neutralization titers compared to 3 mpv, indicating a waning of neutralizing capacity over time.

Notably, the Lambda variant (C.37) was consistently and robustly neutralized by all three donors’ sera at 1 mpv, with ID50 values exceeding those observed for other variants, including the ancestral Wuhan strain. The ID50 values against Lambda reached 1080, 1440, and 4320 for #73, #75, and #85, respectively. However, at 3 mpv, the neutralizing titers decreased by approximately 2- to 8-fold, although the Lambda variant maintained relatively higher titers compared to other variants.

In contrast, Omicron subvariants (including BA.1.18, BA.1.1, BA.4, BA.5, XBB.1) exhibited markedly lower neutralization titers in both 1 mpv and 3 mpv sera. Most ID50 values for these subvariants were below the detection threshold (<45), with the exception of #88, where moderate neutralization was observed against BA.5 and BA.1.18 at 1 mpv (ID50 of 720 and 480, respectively), and residual neutralization was detectable at 3 mpv (ID50 of 180) (Table 3).

Interestingly, the #88 donor, who received a third vaccine dose (booster), demonstrated broader and more sustained neutralization capacity against a wider range of variants, including Omicron subvariants. This suggests that additional vaccination may enhance not only the potency but also the breadth of the humoral immune response, potentially extending cross-variant protection.

The consistently high neutralization titers observed against the Lambda variant (C.37) at 1 mpv in all three donors—surpassing even those elicited against the ancestral Wuhan strain—raised intriguing questions regarding the structural and antigenic properties of this variant. Notably, despite vaccines being based on the Wuhan spike sequence, Lambda virions exhibited heightened susceptibility to neutralization by early-phase polyclonal sera, especially those enriched in IgA and IgM. This paradox prompted us to investigate whether underlying morphological differences between the Lambda and Wuhan strains could explain this enhanced immunogenic accessibility.

To explore this, we employed immuno-NS-EM using both mAb and human serum-derived polyclonal antibodies. Our goal was to visualize the surface structure and antibody binding patterns of both variants under identical labeling conditions. Given the known mutations in the Lambda spike—particularly the RSYLTPGD246–253N deletion in the N-terminal domain and L452Q substitution—previous studies have suggested potential effects on spike density, distribution, or mobility. Such alterations could create structurally favorable conditions for multivalent antibody engagement, particularly by IgM and dimeric IgA, which are highly efficient in cross-linking spatially distributed antigens.

By directly comparing gold-labeled antibody binding and virion size distributions between Wuhan and Lambda strains, we aimed to determine whether spike accessibility and particle morphology may underlie the enhanced neutralization of Lambda observed in functional assays. The following section presents these EM-based structural findings.

### 3.6. Electron Microscopy Reveals Structural Differences and Enhanced Antibody Accessibility in the SARS-CoV-2 Lambda Variant

To further investigate the structural basis underlying the enhanced neutralization observed against the Lambda variant—despite mRNA vaccines being based on the ancestral Wuhan spike—we performed immunogold negative-stain immuno-NS-EM to directly visualize antibody binding and virion morphology. We selected the #88 donor for serum-based imaging due to the following factors: (i) the highest neutralizing activity against LpVspike pseudovirus at 1 mpv (Figure 5d), (ii) a pronounced 2.2-fold decline in ID_50_ titers between 1 mpv and 3 mpv, and (iii) receipt of a third vaccine dose, which provided broader and more sustained neutralization, especially against immune-evasive variants such as Omicron.

Authentic SARS-CoV-2 Wuhan and Lambda virions were incubated either with 1 mpv or 3 mpv vaccinated human serum, or with human monoclonal anti-SARS-CoV-2 IgG, IgA, or IgM antibodies, all derived from the 8A5 clone targeting the Wuhan spike. These antibodies were commercially sourced from Elabscience, ensuring a shared Fab domain and thus epitope uniformity across isotypes. This approach provided a standardized tool for dissecting the contribution of antibody isotype and valency to virion surface engagement.

**Figure 7:**
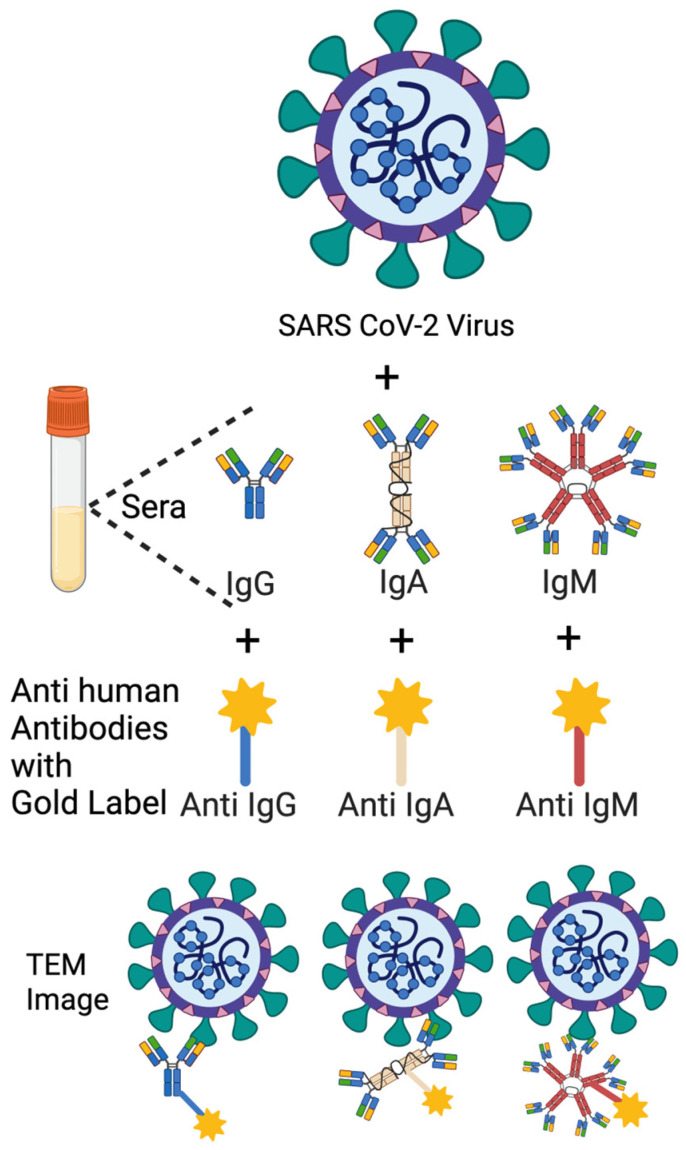
The illustration of the immuno-NS-EM method for each mAbs. An amount of 100 µL TCID50 1.58 × 10^6^ Wuhan or TCID50 3.41 × 10^6^ SARS-CoV-2 TY33-456 lambda variant from −80 °C was mixed with 10 µL 1 mpv and 3 mpv sera at 37 °C by 1 h, and then the virus + sera complex was incubated with 1:20 rate 4 nm Colloidal Gold Anti-Human IgG or IgA or IgM separately for each at room temperature by half an hour. For the control experiment, Virus + Anti-Human IgM or IgA or IgG Gold Label mixture also incubated at room temperature for half an hour. This mixture was fixed by 1:1 with %2.5 glutaraldehyde, placed on carbon-covered grids and stained with 2% phosphotungstic acid and observed by EM. Figure 7 was created in BioRender.

**Figure 8:**
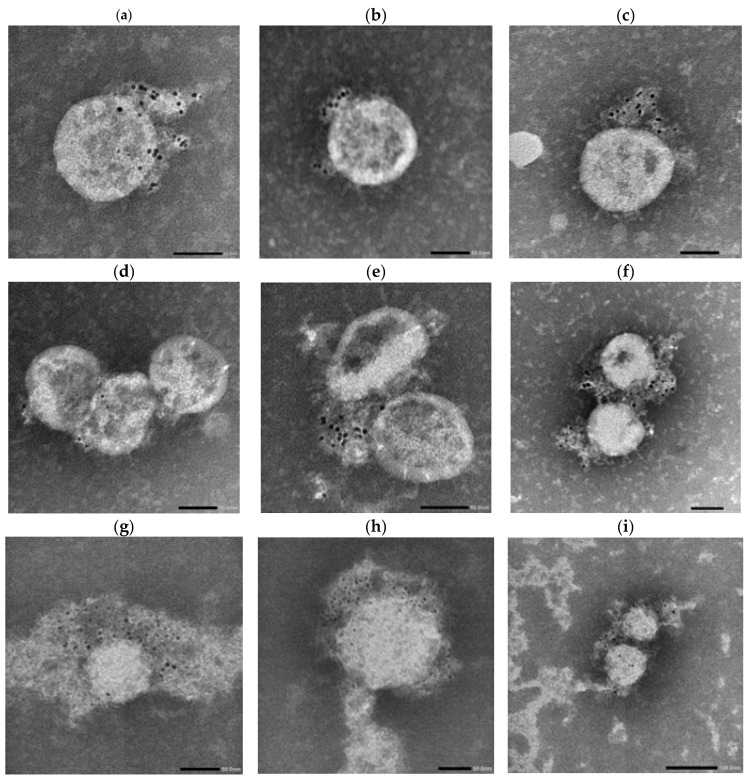
Immunoblot EM imaging of SARS-CoV-2 Wuhan virions with anti-SARS-CoV-2 human monoclonal antibodies. Transmission electron microscopy images of authentic SARS-CoV-2 Wuhan virions incubated with human anti-SARS-CoV-2 monoclonal IgA (**a**–**f**) antibodies or vaccinated human sera (**g**–**i**) and labeled with 4 nm Colloidal Gold Anti-Human IgG or IgM separately (black dots). (**a**–**c**) Wuhan strain; (**d**–**f**) Lambda variant. Scale bars: (**a**–**h**) = 50 nm. (**i**) = 100 nm.

**Figure 9:**
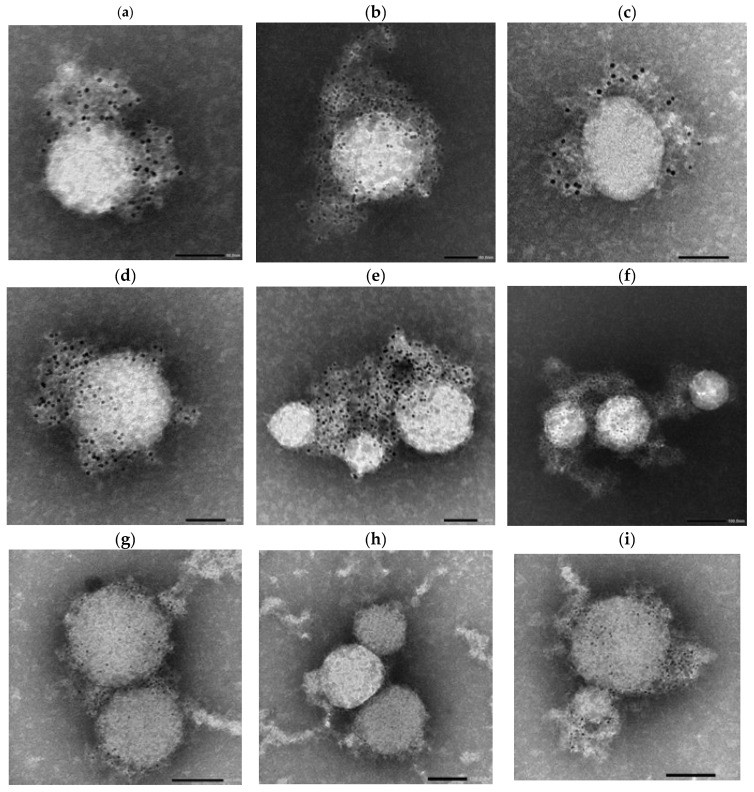
Immunoblot EM imaging of SARS–CoV–2 TY33–456–C7–Lambda virions with anti-SARS-CoV-2 human monoclonal antibodies. Transmission electron microscopy images of authentic SARS–CoV–2 Lambda virions incubated with human anti-SARS–CoV–2 monoclonal IgG (**c**,**d**) or IgM (**a**,**b**,**e**,**f**) antibodies (**a**–**f**) or vaccinated human sera (**g**–**i**) and labeled with 4nm Colloidal Gold Anti-Human IgG or IgM separately (black dots). (**a**–**c**) Wuhan strain; (**d**–**f**) Lambda variant. Scale bars: (**a**–**e**) = 50 nm. (**f**–**i**) = 100 nm.

Following incubation, the antibody–virus complexes were labeled with 4 nm Colloidal Gold-conjugated Anti-Human secondary antibodies specific for IgG, IgA, or IgM and subsequently imaged using NS-EM (Figure 7, Figure 8 and Figure 9).

This experiment was designed to determine whether differences in spike protein organization, virion size, or antigen accessibility between the Wuhan and Lambda variants could contribute to the increased neutralization sensitivity of Lambda observed in early-phase sera. The use of both serum-derived and standardized monoclonal antibodies allowed us to distinguish structural features intrinsic to the virions from donor-specific immune responses.

Across panels, Lambda virions showed more prominent gold labeling, particularly in IgM-labeled samples (Figure 9a,b,e,f), compared to Wuhan virions (Figure 8a–f), which exhibited more punctate and sparsely distributed labeling.

Morphologically, Wuhan virions appeared relatively uniform and spherical with diameters in the range of ~80–120 nm. In contrast, Lambda virions exhibited greater heterogeneity in particle size, with estimated diameters ranging from approximately 50 nm to 200 nm.

In serum-treated panels, Lambda virions again exhibited more visible labeling than Wuhan virions across all tested isotypes (IgG, IgA, IgM), as observed in Figure 9g–i versus Figure 8g–i.

To further understand how virion size heterogeneity may impact isotype-specific binding, we modeled spike spacing and antibody accessibility across virions of varying diameters. The geometric constraints on bivalent or multivalent binding likely influence isotype efficiency.

### 3.7. Size-Dependent Spike Distribution Impacts Isotype Binding

To better understand how virion size and spike density may influence antibody binding, we hypothesized that the multivalent nature of IgA and IgM enables these isotypes to maintain effective binding even when spike proteins are sparsely distributed on the viral surface. This hypothesis is particularly relevant for variants such as Lambda, which exhibit broader virion size heterogeneity and potentially altered spike spacing.

**Figure 10:**
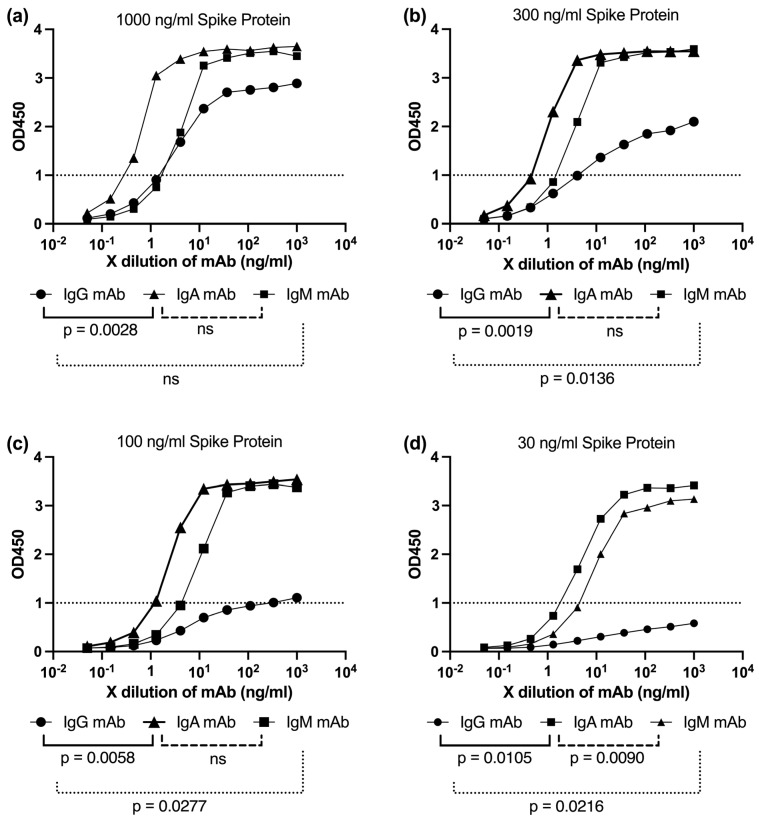
Binding performance of IgG, IgA, and IgM antibodies across varying spike protein densities in ELISA: To simulate antigen density variability due to differences in virion size and spike distribution—such as those observed in the Lambda variant—ELISA plates were coated with serially diluted SARS-CoV-2 trimeric spike protein (1000, 300, 100, and 30 ng/mL). The binding responses of monoclonal IgG, IgA, and IgM antibodies sharing the 8A5 clone same Fab domain were assessed across these antigen concentrations. Each antibody was initially diluted to 1 µg/mL and then serially diluted 10 times in 3-fold steps. Symbols: circles represent IgG, squares represent IgA, and triangles represent IgM. (**a**) OD_450_ absorbance curves for IgG, IgA, and IgM with plates coated at 1000 ng/mL spike protein. (**b**) OD_450_ absorbance curves for IgG, IgA, and IgM with plates coated at 300 ng/mL spike protein. (**c**) OD_450_ absorbance curves for IgG, IgA, and IgM with plates coated at 100 ng/mL spike protein. (**d**) OD_450_ absorbance curves for IgG, IgA, and IgM with plates coated at 30 ng/mL spike protein.

To investigate this, we performed ELISA assays using plates coated with serially diluted spike protein, ranging from 1000 ng/mL to 30 ng/mL (1000, 300, 100, and 30 ng/mL), to simulate varying degrees of antigen availability (Figure 10a–d). The binding capacities of monoclonal IgG, IgA, and IgM antibodies were assessed across these conditions to evaluate their sensitivity to reduced antigen density, mimicking sparse or uneven spike distribution.

IgG binding exhibited a sharp decline as the spike protein concentration decreased, showing limited engagement at 100 ng/mL (Figure 10c) and near-OD1 levels at 30 ng/mL (Figure 10d). In contrast, both IgA and IgM antibodies maintained robust binding even at lower spike densities (Figure 10a–d). This pattern is consistent with their multivalent architecture—dimeric for IgA and pentameric for IgM—which allows simultaneous engagement with multiple epitopes, enhancing avidity and overall retention.

These findings reinforce the hypothesis that the superior neutralization breadth of IgA and IgM, especially against structurally diverse or low-spike-density variants such as Lambda, may stem from their ability to maintain high binding efficiency under conditions of limited antigen availability (Figure 10a–d). This functional advantage is likely relevant in vivo, where spike density and distribution on virions can vary substantially depending on the variant.

## 4. Discussion

This study aimed to understand how the composition and timing of antibody isotypes influence the potency and breadth of neutralizing activity against SARS-CoV-2 variants following mRNA vaccination. By evaluating sera collected one month (1 mpv) and three months (3 mpv) post-vaccination, we assessed not only quantitative differences in neutralization but also the isotype dynamics and structural correlates underlying these immune responses.

Our findings suggest that, in the tested samples, sera collected at 1 mpv generally exhibited stronger and broader neutralizing activity against most SARS-CoV-2 variants, including the ancestral Wuhan strain, Alpha, and Gamma, compared to sera collected at 3 mpv. Notably, in the single donor who received three vaccine doses (sample #88), the 1 mpv sera also showed higher neutralization against most variants. However, unlike the two-dose samples, #88 was able to neutralize Omicron variants at both 1 mpv and 3 mpv (Figure 4). This observation suggests that increasing the number of vaccine doses may broaden the antibody repertoire and confer additional protection against antigenically divergent variants. Nevertheless, as this finding is based on a single case, it should be interpreted with caution.

Among all variants tested, the Lambda variant exhibited a particularly striking neutralization profile, with ID_50_ values consistently exceeding those observed for other variants and even surpassing the ancestral Wuhan strain. This unexpected sensitivity prompted further structural investigation into the virion-level features of Lambda. Although our data suggest that the enhanced susceptibility of Lambda may reflect altered virion morphology and spike spacing, it is also possible that other factors, such as intrinsic antigenic properties, differences in infectivity, or altered receptor-binding affinity, contributed to this observation. Further studies will be needed to definitively confirm the underlying mechanisms.

Isotype profiling revealed a general decline in IgG, IgA, and IgM levels over time in #73, #75, and #83 (Figure 6a–c). However, #88 maintained relatively stable IgG levels between 1 and 3 mpv, while IgA and IgM levels declined markedly (Figure 6d). This differential isotype kinetics may explain the preserved IgG-mediated neutralization in #88 at later timepoints, though the loss of multivalent IgA and IgM likely diminished breadth, particularly against variants such as Lambda. It is also plausible that broader neutralization in some individuals, such as #88, reflects not only preserved IgG levels but also features of hybrid immunity or enhanced immune imprinting from prior exposure.

The superior neutralization observed at 1 mpv likely reflects the enhanced avidity conferred by IgA and IgM. These isotypes can simultaneously engage multiple epitopes—even when the affinity of individual Fab arms is compromised by spike mutations—resulting in increased neutralization efficiency. This is consistent with prior reports showing that multimeric IgA and IgM antibodies engineered with identical Fab domains to IgG neutralize SARS-CoV-2 125- to 225-fold more potently [14,15,34]**.**

Our time-matched analysis supports previous observations that IgA dominates the early-phase neutralizing response, while IgG becomes more prominent over time stages [35]. Notably, live-virus assays showed that the Lambda variant was robustly neutralized by 1 mpv sera, especially from #75, #83, and #88, indicating a potential link between early IgA/IgM activity and effective Lambda neutralization.

To investigate the structural basis underlying these functional differences, we performed immunogold electron microscopy. Comparative imaging of Wuhan and Lambda virions revealed clear differences in spike accessibility and virion morphology. Lambda virions exhibited a broader size distribution (50–200 nm) and higher density of gold-labeled IgM binding, suggesting enhanced epitope exposure or altered spike arrangements conducive to multivalent antibody engagement. This structural profile may enhance the binding efficiency of IgM, especially on smaller virions with fewer spikes, where multivalent binding can saturate available epitopes more effectively.

Increased IgM labeling on Lambda virions likely reflects the advantage of pentameric structure in mediating high-avidity interactions. Conversely, larger virions with expanded surface areas may require higher antibody concentrations for comparable coverage. These findings correspond with functional neutralization data showing superior Lambda inhibition by 1 mpv sera enriched in IgM and IgA.

Serum-derived antibodies from #88 also displayed enhanced binding to Lambda virions, suggesting that both monoclonal and polyclonal responses are shaped by variant-specific structural features. These observations emphasize the influence of virion morphology and spike organization on antibody recognition and support strategies aimed at preserving multivalent responses in future vaccines.

Kimura et al. (2022) reported that the Lambda spike harbors a deletion (RSYLTPGD246–253N) in the N-terminal domain, which may disrupt local spike architecture and reduce the accessibility of certain neutralizing IgG epitopes in pseudovirus-based assays [36]. Interestingly, this structural rearrangement, while potentially limiting monovalent IgG engagement, could conversely promote enhanced binding by multivalent antibodies such as IgA and IgM due to increased spacing between spike trimers. This interpretation is supported by our findings that early-phase sera, enriched in IgA and IgM, demonstrated potent Lambda neutralization and sustained binding under low-density spike conditions in ELISA experiments. Therefore, our observations is not inconsistent with previous reports but rather highlight how spike conformational changes can differentially impact neutralization depending on antibody isotype and valency.

Electron microscopy further revealed that the Lambda variant exhibits a broader virion size distribution (ranging from 50 to 200 nm in Figure 9) compared to the ancestral Wuhan strain (80–120 nm in Figure 8). This structural heterogeneity likely introduces greater variability in inter-spike distances on the viral surface, potentially affecting the accessibility and cooperative binding of multivalent antibodies.

Beyond isotype composition, it is also important to recognize that neutralization assays may not fully capture the contribution of Fc-dependent effector functions.

To functionally probe this concept, we conducted spike titration ELISA experiments using plates coated with decreasing concentrations of trimeric spike protein (Figure 10). The aim was to simulate varying antigen densities that might reflect the differential spatial arrangements of spikes on Lambda versus Wuhan virions. These experiments revealed that IgA and IgM maintained stronger binding responses compared to IgG under conditions of reduced spike availability. This observation supports the hypothesis that the higher valency of IgA and IgM allows them to engage multiple epitopes effectively, even when spike density is low or unevenly distributed on the virion surface. However, ELISA performed on flat surfaces may not fully capture the three-dimensional organization and mobility of spike proteins on intact virions. Therefore, the observed binding patterns may not entirely reflect the complexity of in vivo antigen presentation. Thus, our ELISA findings provide additional supportive evidence for the superior neutralization breadth exhibited by IgA and IgM antibodies against antigenically and morphologically distinct viral variants such as Lambda.

Such an arrangement may enhance the ability of multivalent antibodies—such as dimeric IgA and pentameric IgM—to cross-link multiple spikes. This is supported by Figure 9, where Lambda virions incubated with monoclonal IgM exhibit dense, clustered gold labeling, indicating efficient multivalent engagement. This structural adaptation may enhance neutralization at early timepoints. In contrast, reduced IgA and IgM levels in 3 mpv sera may diminish this advantage, particularly against larger, more complex Lambda particles.

Collectively, our findings suggest that early-phase multivalent antibody responses—particularly IgA and IgM—may contribute importantly to effective and broad neutralization against emerging SARS-CoV-2 variants. The structural features of variants like Lambda may render them more susceptible to such responses, offering insights into antigen design and temporal optimization of vaccine-induced immunity. These results advocate for next-generation vaccine strategies that preserve or reinduce early-phase isotype diversity to ensure durable, cross-variant protection.

## 5. Limitations

This study included a limited number of donors and variants, which may affect generalizability. Nevertheless, observed trends were consistent, and higher-resolution imaging could further strengthen these findings.

## Figures and Tables

**Table 1:** SARS-CoV-2 strains used in this study and their infectious titers. This table summarizes the strain names, Pango lineages, GISAID identifiers, and TCID_50_/mL values determined by endpoint dilution assays for the SARS-CoV-2 variants included in the neutralization experiments.

Strain Name	Pango Lineage	Strain Name	GISAID ID	TCID50
Wuhan	A	2019-nCoV/Japan/TY/WK-521/2020	EPI_ISL_408667	1.58 × 10^6^
QK001	B.1.1.7	hCoV-19/Japan/QK001/2020	EPI_ISL_9065387	5.00 × 10^5^
QHN001	B.1.1.7	hCoV-19/Japan/QHN001/2020	EPI_ISL_804007	2.32 × 10^6^
QHN002	B.1.1.7	hCoV-19/Japan/QHN002/2020	EPI_ISL_804008	2.32 × 10^6^
TY8-612	B.1.351	hCoV-19/Japan/TY8-612/2021	EPI_ISL_1123289	3.41 × 10^6^
TY7-501	P.1	hCoV-19/Japan/TY7-501/2021	EPI_ISL_833366	2.32 × 10^6^
TY7-503	P.1	hCoV-19/Japan/TY7-503/2021	EPI_ISL_877769	5.00 × 10^5^
TY11-927	AY.122	hCoV-19/Japan/TY11-927/2021	EPI_ISL_2158617	5.00 × 10^5^
TY33-456	C.37	hCoV-19/Japan/TY33-456/2021	EPI_ISL_4204973	3.41 × 10^6^
TY28-444	P.3	hCoV-19/Japan/TY28-444/2021	EPI_ISL_3869208	1.58 × 10^5^
TY26-717	B.1.621	hCoV-19/Japan/TY26-717/2021	EPI_ISL_4470503	2.32 × 10^6^
TY38-873	BA.1.18	hCoV-19/Japan/TY38-873/2021	EPI_ISL_7418017	2.32 × 10^6^
TY38-871	BA.1.1	hCoV-19/Japan/TY38-871/2021	EPI_ISL_7571618	2.32 × 10^6^
TY41-703	BA.4	hCoV-19/Japan/TY41-703/2022	EPI_ISL_13278440	7.34 × 10^5^
TY41-702	BA.5	hCoV-19/Japan/TY41-702/2022	EPI_ISL_13241867	2.32 × 10^6^
TY41-686	XE	hCoV-19/Japan/TY41-686/2022	EPI_ISL_12703378	1.08 × 10^6^
TY41-795	XBB.1	hCoV-19/Japan/TY41-795/2022	EPI_ISL_15669344	2.32 × 10^6^
TY41-796	BA.5.11111	hCoV-19/Japan/TY41-796/2022	EPI_ISL_15579783	5.00 × 10^5^

**Table 2:** ID50 values of individual mRNA-vaccinated sera against LpVspike. ID50 titers were determined based on neutralization curves shown in Figure 1.

Higher ID50 Sera											
**Sera**	**#2**	**#1**	**#6**	**672**	**#4**	**IgMsera**	**710**	**783**	**#5**	**#10**	685
ID50	2280	1440	1440	1440	1440	1403	1350	1200	1130	1100	1050
**Intermediate ID50 Sera**											
Sera	#12	#3	655	713	#7	#8	684	664	IgGsera		
ID50	973	950	720	720	616	616	600	600	521		
**Lower ID50 Sera**											
Sera	#11	667	843	784	797	749	598	#13	675		
ID50	360	260	180	180	138	114	90	68	45		

**Table 3:** Neutralization titers (ID50) and antibody isotype levels (OD_1_) of mRNA-vaccinated sera against LpVspike. ID50 values were obtained from the neutralization assay results shown in Table 2**,** while OD_1_ values for IgG, IgA, and IgM were derived from the ELISA data presented in Figure 3.

Human Sera	#2	672	#6	#1	710	783	#3	684	675
ID50	2280	1440	1440	1440	1350	1200	950	600	45
OD1 for IgG	>312,500 (618,000)	>218,500 (547,000)	297,360	172,140	218,700	132,100	261,610	258,000	1800
OD1 for IgA	5242	8100	6680	1741	2400	280	4680	5550	N/C
OD1 for IgM	1000	400	N/C	664	635	837	600	N/C	N/C

**Table 4:** ID50 (ng/mL) values of authentic SARS-CoV-2 variants tested against sera collected from Biontech-vaccinated individuals at 1 mpv and 3 mpv. #75 and #83 donors received two vaccine doses, whereas #88 received three doses. Sera were serially diluted starting from 1:45, with nine two-fold dilutions and four replicate wells per dilution. Neutralization activity was determined based on CPE. The gray-shaded cells in Figure 4 indicate ID50 values for variants where neutralization was higher/equal at 1 mpv compared to 3 mpv.

Strain Name	Clade	Pango Lineage	#75			#83			#88		
			1 mpv ID50	3 mpv ID50	X Fold	1 mpv ID50	3 mpv ID50	X Fold	1 mpv ID50	3mpv ID50	X Fold
Wuhan	Progenitor	A	360	90	4	135	67.5	2	1200	720	1.7
QK001	α Alpha	B.1.1.7	180	<45	4	270	90	3	2400	1200	2
QHN001	α Alpha	B.1.1.7	360	90	4	254	113	2.3	1440	1200	1.2
TY8-612	β Beta	B.1.351	135	<45	3	56	<45	1.2	720	540	1.3
TY7-503	γ Gamma	P.1	135	<45	3	67.5	<45	1.5	960	480	2
TY11-927	δ Epsilon	AY.122	720	360	2	180	113	1.6	2160	720	3
TY33-456	λ Lamda	C.37	1080	720	1.5	1440	180	8	4320	1080	4
TY28-444	Ɵ Theta	P.3	270	90	3	113	<45	3	1080	1080	1
TY26-717	µ Mu	B.1.621	180	90	2	143	<45	3.2	1920	1080	1.8
TY38-873	Omicron	BA.1.18	<45	<45	N/C	<45	<45	N/C	480	180	2.7
TY38-871	Omicron	BA.1.1	<45	<45	N/C	<45	<45	N/C	1200	1080	1.1
TY41-703	Omicron	BA.4	<45	<45	N/C	<45	<45	N/C	240	360	0.7
TY41-702	Omicron	BA.5	<45	<45	N/C	<45	<45	N/C	720	180	4
TY41-686	Omicron	XE	<45	<45	N/C	<45	<45	N/C	480	600	0.8
TY41-795	Omicron	XBB.1	<45	<45	N/C	<45	<45	N/C	135	135	1
TY41-796	Omicron	BA.5.11111	<45	<45	N/C	<45	<45	N/C	180	60	3

## Data Availability

The data presented in this study are available on request from the corresponding author. The data are not publicly available due to privacy or ethical restrictions.
